# A Simple Yet Effective Whole-Body Locomotion Framework for Quadruped Robots

**DOI:** 10.3389/frobt.2020.528473

**Published:** 2020-11-19

**Authors:** Gennaro Raiola, Enrico Mingo Hoffman, Michele Focchi, Nikos Tsagarakis, Claudio Semini

**Affiliations:** ^1^Dynamic Legged Systems Lab, Istituto Italiano di Tecnologia, Genoa, Italy; ^2^Humanoid and Human Centred Mechatronics Lab, Istituto Italiano di Tecnologia, Genoa, Italy

**Keywords:** legged robots, planning, optimization, whole-body control, locomotion framework

## Abstract

In the context of legged robotics, many criteria based on the control of the Center of Mass (CoM) have been developed to ensure a stable and safe robot locomotion. Defining a whole-body framework with the control of the CoM requires a planning strategy, often based on a specific type of gait and a reliable state-estimation. In a whole-body control approach, if the CoM task is not specified, the consequent redundancy can still be resolved by specifying a postural task that set references for all the joints. Therefore, the postural task can be exploited to keep a well-behaved, stable kinematic configuration. In this work, we propose a generic locomotion framework which is able to generate different kind of gaits, ranging from very dynamic gaits, such as the trot, to more static gaits like the crawl, without the need to plan the CoM trajectory. Consequently, the whole-body controller becomes planner-free and it does not require the estimation of the floating base state, which is often prone to drift. The framework is composed of a priority-based whole-body controller that works in synergy with a walking pattern generator. We show the effectiveness of the framework by presenting simulations on different types of simulated terrains, including rough terrain, using different quadruped platforms.

## 1. Introduction

Over the last few years in the context of legged robots, a lot of effort has been devoted to designing controllers and planners for locomotion. However, most of the time these two elements are considered separately (Kalakrishnan et al., 2010; Winkler et al., 2015; Fankhauser et al., 2016). Typically the controller requires that the trajectory of the CoM is specified to ensure the stability during locomotion^1^. In a different manner from the task that controls the orientation of the base, for which an Inertial Measurement Unit (IMU) can provide reliable measurements, the planning and tracking of the CoM requires a state-estimation algorithm to obtain its linear position and velocity (Bloesch et al., 2012; Nobili et al., 2017). Even though these algorithms achieve good results by fusing different sources (e.g., leg odometry, vision, and inertial measurements) their estimation has the potential to drift due to bias in the sensors, feet slippage, visual occlusions and compliance of the mechanical structure. Moreover, designing trajectories for the CoM is not a trivial task, because, despite satisfying stability constraints, consideration must be taken of the specific kinematic properties of the robot beforehand. For instance, a certain CoM position could correspond to an undesirable kinematic configuration: close to the kinematic limits of the robot, and/or with low leg mobility (Focchi et al., 2020). To avoid inconvenient kinematic configurations while walking, it is crucial to provide enough mobility and prevent progressive degeneration of the support polygon and thus also keep the joint efforts limited. As a matter of fact, if the support polygon shrinks, the robustness decreases because the legs can lose mobility for future steps. Differently from the trunk orientation task, where the reference orientation usually does not change so frequently (and in general for common gaits like walk and trot is bounded in a way that the joints are always inside their limits), planning a feasible trajectory for the CoM requires a more complex procedure, often involving numerical optimization techniques.

### 1.1. Related Work

**Figure 1:**
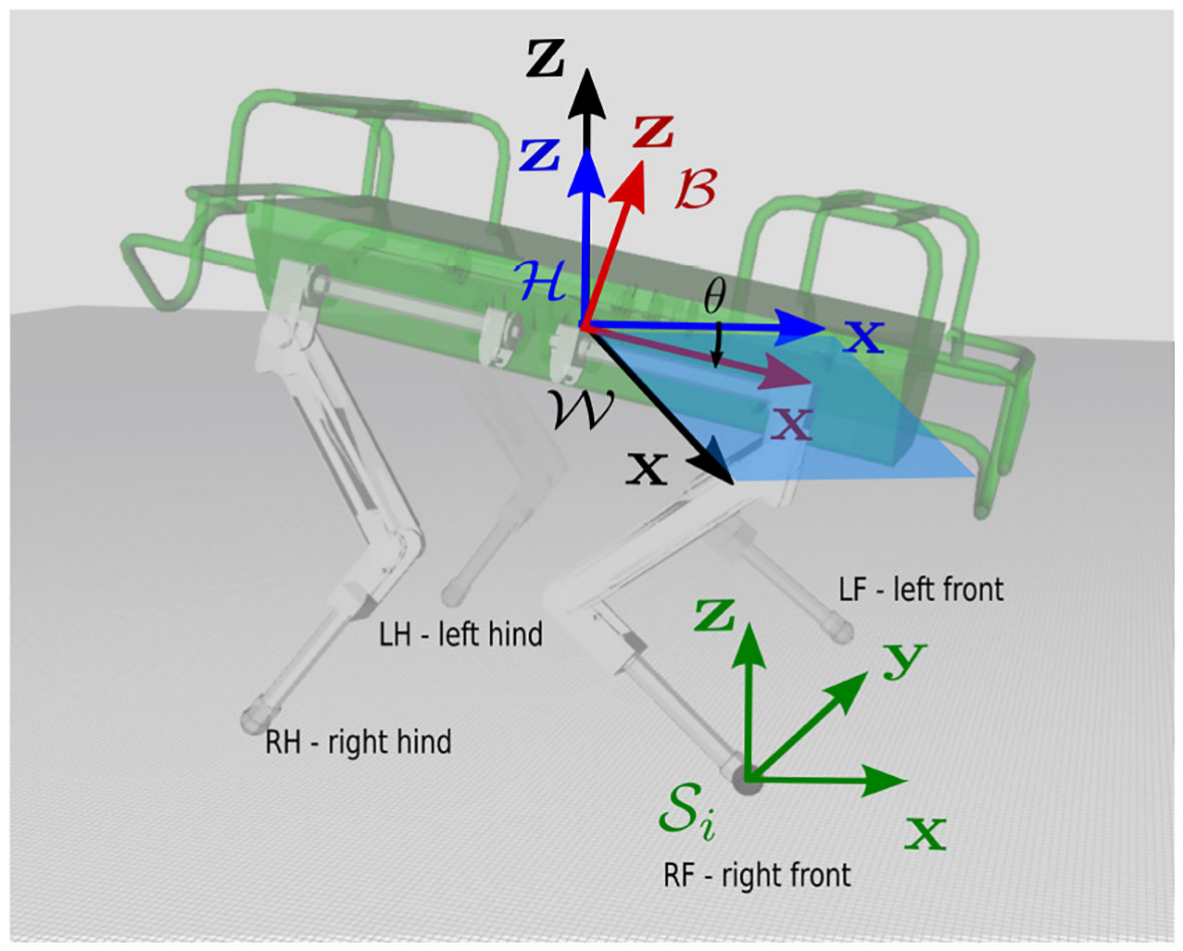
The figure shows the horizontal frame H, placed at the base link *bl* but aligned with gravity. The *X*-axes of the world and of the horizontal frame are co-planar but with different yaw orientations. The swing frame $S_{i}$ for the right front leg of the robot, is located at the foot and is aligned with the horizontal frame (on flat terrain). θ is the pitch of the base link.

Priorities are a strategy to deal with conflicting tasks where some of them are more critical. This strategy ensures the achievement of high priority tasks at the expense of other tasks with lower priority. In robotics, hierarchical approaches based on priorities were originally introduced for inverse kinematics problems with the works of Whitney (1969) and Liegeois (1977), and successively by Nakamura et al. (1987) and Siciliano and Slotine (1991). Khatib (1987) also implemented them for inverse dynamics control of redundant manipulators involving two task levels: a first level to control the position of the end-effector and another to control the redundant joints. Later on, Sentis and Khatib (2004) extended this approach to humanoid robots in contact with the environment with an arbitrary number of tasks. However, all these works are projection-based^2^ and do not allow the enforcement of *explicitly inequality* constraints. On the other hand, the tasks' completion is often bounded by the robot's workspace and its own technological limits that are typically expressed as inequality constraints (e.g., joint limits, actuation limits, and friction limits). To take inequality constraints into account, optimization techniques have been introduced to cast the control problem as an optimization problem (Decŕe et al., 2009; De Lasa and Hertzmann, 2009; Righetti et al., 2011; Saab et al., 2013). In these approaches, the robot dynamics can be imposed as an *equality* constraint to ensure a physically consistent evolution of the robot state variables. Given the quadratic nature of the cost functions involved^3^ and the linearity of the constraints, to render the inverse dynamics controller, the resulting optimization problem is typically expressed as a Quadratic Program (QP). Alternatively Wensing and Orin (2013) avoided the linear approximation of the friction cones and encoded them as Second Order Cones leading to the SOQP formulation. More recently, *hard-priorities* have been introduced for these inverse dynamics formulations and different efficient implementations have been proposed by Del Prete et al. (2015) and Herzog et al. (2016). Herzog was the first to demonstrate with experiments on humanoid robots the validity of this approach and later the approach was extended on quadruped robots by Bellicoso et al. (2016). Koolen et al. (2016) implemented *soft-priorities* and extensively tested this approach on humanoid robots at the Darpa Robotics Challenge (DRC). Salini et al. (2011) also implemented soft-priorities providing an effective way to avoid torque discontinuities when the relative importance of tasks was modified or when constraints appeared or disappeared. This made their controller able to adapt to dynamically changing environments. The advantage of hard-priorities is that they ensure a perfect achievement of the highest priority task at the price of a high computational time (e.g., one QP is solved for each priority level), but it can happen that there is no redundancy left to achieve lower priority tasks. Indeed strict priorities can be sometimes so conservative that they may completely block lower-priority tasks. On the other hand, with soft-priorities the program is solved only once, and the computation time mainly depends on the dimension of the set of constraints. However, it is not always easy to define the relative weights for the tasks because a good trade-off must be found between the different terms in the cost function. In addition a scaling of the weights must be considered to account for the different size of the SI units in the cost variables. Finally, the authors have recently proposed the formalization of prioritized whole-body Cartesian impedance control as a cascade of QPs (Hoffman et al., 2018) together with the post-optimization of contact forces (Laurenzi et al., 2019) in the case of floating-base systems. All these approaches require the specification of the CoM task in (at least) the *X* and *Y* directions (see Figure 1 for frame definition).

### 1.2. Proposed Approach and Contribution

**Figure 2:**
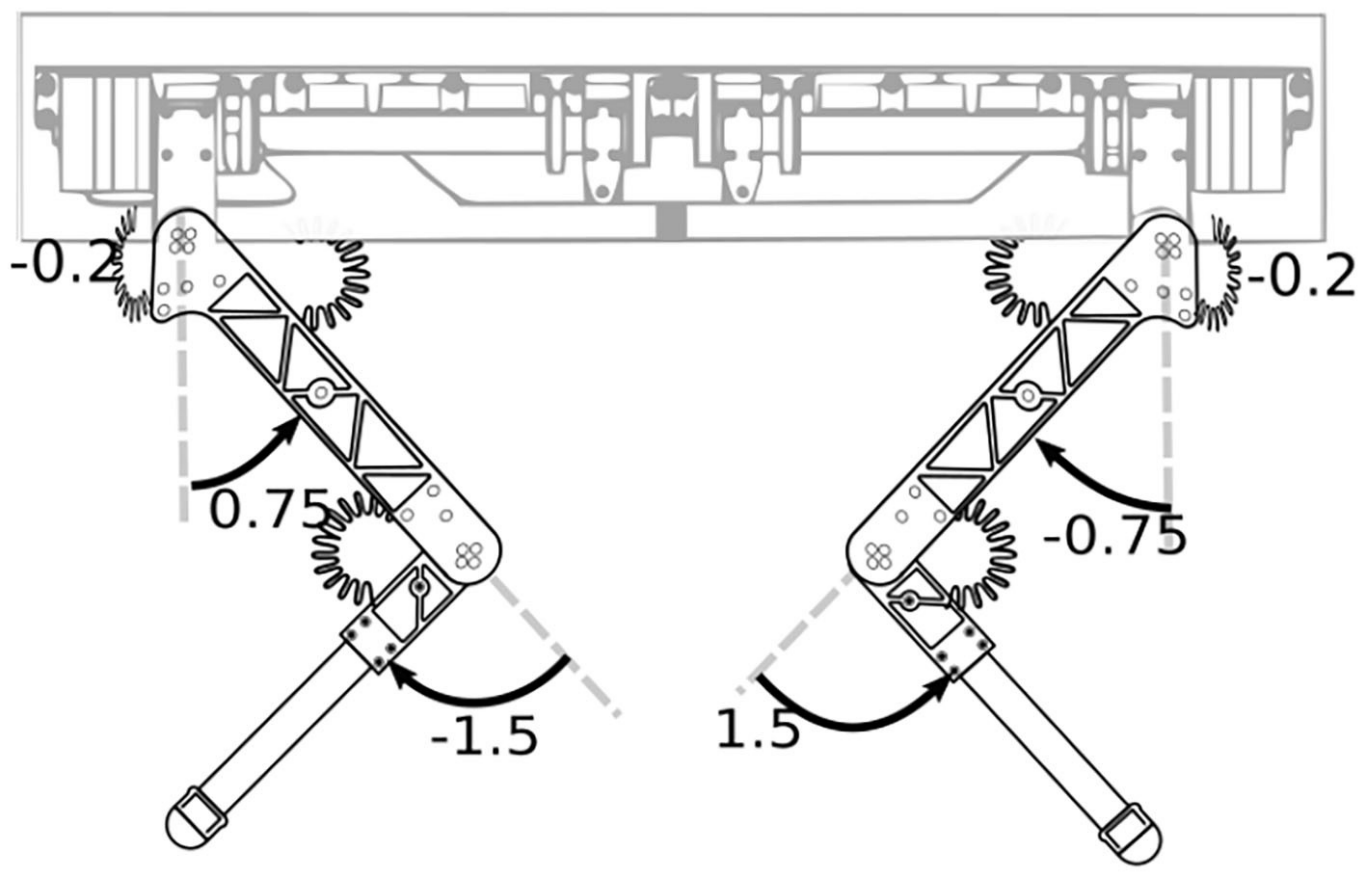
Nominal configuration used as reference for the postural task (values shown in radians).

Our approach builds on top of previous works (Hoffman et al., 2018; Laurenzi et al., 2019) on hierarchical Cartesian impedance control with QP optimization. We extended these works by proposing a novel locomotion *framework* that: (1) avoids the specification of a task for the CoM both in terms of planning and control, making the framework *planner-free*, (2) consequently, does not require inputs from a state-estimation algorithm; (3) keeps the robot in a kinematically “appealing” configuration (e.g., far from joint limits, with a good leg mobility); (4) is robust on rough terrain. The approach achieves a synergy between the planner and the controller by exploiting the *hierarchical* nature of our whole-body optimization. Referring to Table 1 for the priority order of the tasks, we placed the postural task at the lowest priority. The postural task will exploit the Degrees of Freedom remaining from the higher priority tasks to keep the robot close to a preferable *nominal* kinematic configuration (see Figure 2). This generates a connection between the motion of the trunk and the location of the contacts. Therefore, the postural task acts as a set of “elastic linkages,” and determines the *linear* motion of the base, aligning that with the feet while trying to maintain a “nominal” configuration of the robot. Consequently, it eliminates the complexity of designing a CoM trajectory that takes into account the changing shape of the support polygon during locomotion, making the proposed approach planner-free. The locomotion is driven by a terrain-consistent (haptic) *stepping strategy* that selects the footholds to realize a desired velocity command for the robot base. Instead, the orientation of the base is controlled in a separate task at a *higher* priority and will be accommodated by the postural task being this at *lower* priority level. The price to pay for the absence of the CoM task is that the locomotion stability is no longer guaranteed [e.g., the Zero Moment Point (ZMP) could end-up on the boundary of the support polygon]. However, this is not a big issue for quadrupeds, if the swing phases are fast enough (Sproewitz et al., 2011).

**Table 1:** Order of priorities.

**Task name**	**Symbol**	**Priority**
Contact task	${\;}^{W}T_{c_{i}}^{\to }$	1
Trunk orientation task	${\;}^{W}T_{bl}^{∠}$	2
Postural task	$T_{\ddot{q}}$	3

To summarize, the contributions of the present work are:

- A *planner-free* locomotion framework for rough terrain that can be implemented in *real-time*. The framework is composed of a hierarchical whole-body inverse dynamics optimization and a walking pattern generator for omni-directional motions. It can handle different gaits, such as crawl and trot, and requires only desired base velocities as high-level inputs from the operator. It does not require the specification of any CoM task and it allows to decouple the base orientation from the generation of the swing trajectories.
- an *experimental* contribution where we demonstrate the effectiveness of our locomotion framework in simulation on different quadruped platforms, such as Hydraulically actuated Quadruped (HyQ) and ANYmal (ANYmal). Preliminary results were carried out on the real HyQ platform.

### 1.3. Outline

The rest of this paper is structured as follows: in section 2 we describe the Hierarchical Whole-Body Operational Space formulation that we use to enforce priorities in our locomotion framework. In section 3 we present the walking pattern generator while section 4 discusses some details useful for the implementation of the whole-body framework on the real robot. In section 5 we present both simulation and preliminary experimental results, and we conclude with section 7.

## 2. Whole-Body Operational Space Controller

In this section we introduce the formulation of the *Whole-Body Operational Space Controller* approach we developed.

### 2.1. Model, Tasks, and Constraints

We describe the configuration of our robotic system using *f* + *n* joint variables:

(1) $$\begin{array}{l} \text{q}=\left[\begin{array}{c} {\text{q}}_{u}\\ {\text{q}}_{a} \end{array}\right], \end{array}$$

where the values of the first *f* virtual joints **q**_*u*_ represent the pose of the (under-actuated) floating-base, using a particular parameterization for the orientation in *SO*(3)^4^ and the last *n* = 12 are the angular positions of the actuated joints (${\text{q}}_{a}\in {\mathbb{R}}^{n}$). We describe with $\overset{\cdot }{q}={\left[{\overset{\cdot }{q}}_{u}^{T}\;{\overset{\cdot }{q}}_{a}^{T}\right]}^{T}\in {\mathbb{R}}^{6+n}$ the vector of generalized velocities; the linear and angular velocities of the base, ${\text{v}}_{fb}\in {\mathbb{R}}^{6}$, are provided by a proper floating-base Jacobian:

(2) $$\begin{array}{l} {\text{J}}_{fb}{\overset{\cdot }{q}}_{u}={\text{v}}_{fb}. \end{array}$$

The dynamic model of the floating-base system in contact with the environment is given by the following:

(3) $$\begin{array}{l} \text{M}\left(\text{q}\right)\ddot{q}+\text{h}\left(\text{q},\overset{\cdot }{q}\right)={\text{S}}^{T}\tau +{\text{J}}_{c}{\left(\text{q}\right)}^{T}{\text{F}}_{c}. \end{array}$$

According to our parameterization, **M** ∈ ℝ^(6+*n*) × (6+*n*)^ is the joint space inertia matrix, $\ddot{q}\in {\mathbb{R}}^{n+6}$ are the joint accelerations, **h** ∈ ℝ^*n*+6^ are torques which account for non-linear terms in the dynamics, **S** = [0_*n*×6_*I*_*n*×*n*_] is a selection matrix that accounts for the fact that the floating-base is not actuated, ***τ*** ∈ ℝ^*n*^ are the actuated torques, finally ${\text{J}}_{c}\in {\mathbb{R}}^{3c\times \left(n+6\right)}$ are the Jacobians of the contacts^5^ and ${\text{F}}_{c}\in {\mathbb{R}}^{3c}$ is the vector of contact forces expressed in the world W frame, where *c* is the number of contacts. We will enforce in our Whole-Body Control (WBC) formulation, the first six rows of (3) as a constraint $C_{fb}$ to have accelerations $\ddot{q}$ and contact forces **F**_*c*_ consistent with the dynamics of the system:

(4) $$\begin{array}{l} C_{fb}:\;{\text{M}}_{fb}\ddot{q}+{\text{h}}_{fb}={\text{J}}_{c,fb}^{T}{\text{F}}_{c}. \end{array}$$

where ${\text{M}}_{fb}\in {\mathbb{R}}^{6\times n+6}$, ${\text{h}}_{fb}\in {\mathbb{R}}^{6}$ and ${\text{J}}_{c,fb}\in {\mathbb{R}}^{3c\times 6}$ are the first six rows extracted from (3).

In this work, we aim to simplify the gait generation and to make the whole-body controller independent, as much as possible, from the floating-base state estimation. In order to do so, the world frame W origin will always be attached to the base origin and we express the orientation tasks w.r.t. this frame. Additionally for the generation of Cartesian feet trajectories (see section 3.2) we define the H frame. This is the same as the base frame *B* (e.g., moves with the robot) but with the *Z* axis parallel to gravity, i.e., aligned to the inertial frame W (see Figure 1). This frame is also known as *horizontal frame* (Barasuol et al., 2013; Focchi et al., 2020). The base frame and horizontal frames can be extrapolated from IMU measurements.

In our whole-body formulation we intend to consider generalized accelerations and contact forces as optimization variables. Therefore, a generic Cartesian (6D) acceleration should be expressed in terms of these variables as:

(5) $$\begin{array}{l} ẍ=\text{J}\left(\text{q}\right)\ddot{q}+\overset{\cdot }{J}\left(\text{q},\overset{\cdot }{q}\right)\overset{\cdot }{q}, \end{array}$$

where ***ẍ*** ∈ ℝ^6^ is a Cartesian acceleration, **J** is the task Jacobian and the term $\overset{\cdot }{J}\overset{\cdot }{q}\in {\mathbb{R}}^{6}$ accounts for the accelerations due to joint velocities. We can define a Cartesian tracking task for (5) as a quadratic cost function T:

(6) $$\begin{array}{l} \begin{array}{c} T:\;\|\text{J}\ddot{q}-{\ddot{x}}_{r}{\|}_{\text{W}}^{2},\\ {\ddot{x}}_{r}={\ddot{x}}_{d}-\overset{\cdot }{J}\overset{\cdot }{q}+{\text{K}}_{P}\left({\text{x}}_{d}-\text{x}\right)+{\text{K}}_{D}\left({\dot{x}}_{d}-\dot{x}\right), \end{array} \end{array}$$

where ${\ddot{x}}_{r}\in {\mathbb{R}}^{6}$ is a reference Cartesian acceleration vector composed by the feed-forward terms ${\ddot{x}}_{d}-\overset{\cdot }{J}\overset{\cdot }{q}\in {\mathbb{R}}^{6}$ and the feedback terms ${\text{K}}_{P}\left({\text{x}}_{d}-\text{x}\right)+{\text{K}}_{D}\left({\dot{x}}_{d}-\dot{x}\right)\in {\mathbb{R}}^{6}$ that aim to drive the position and velocity tracking error to zero, ${\text{K}}_{P},{\text{K}}_{D}\in {\mathbb{R}}^{6\times 6}$ are positive definite feedback gains matrices. The proper computation of the orientation error between the two poses will be explicitly discussed later on in this section. The matrix **W** ∈ ℝ^6×6^ is the weight matrix associated to the cost function^6^.

Considering the generic Cartesian acceleration task in (6), we can define the *contact task* ${\;}^{W}T_{c_{i}}^{\to }$ for each foot *i* in contact:

(7) $$\begin{array}{l} \begin{array}{c} {\;}^{W}T_{c_{i}}^{\to }:\;\|{\text{J}}_{c,i}\ddot{q}-{\ddot{x}}_{r}{\|}_{{\;}_{\text{W}}}^{2},\\ {\ddot{x}}_{r}=-{\overset{\cdot }{J}}_{c,i}\overset{\cdot }{q}. \end{array} \end{array}$$

In the contact task we set the reference acceleration to be zero (i.e., the feet in contact do not move). The task is defined w.r.t. the world frame W while the superscript → means that only the position part of the task is considered (because we have point feet assumption). We do not set any feedback gain at this level because we do not want to have dependency on the state estimation, that is often prone to drift^7^, while the inter-feet distance will be ensured by the postural task. Contact tasks at the feet are needed to ensure the emergence of the contact forces in (4) needed to compensate for the gravity load acting on the floating base of the robot.

We consider another Cartesian task ${\;}^{W}T_{bl}^{∠}$ to control the orientation of the *base* of the robot:

(8) $$\begin{array}{l} \begin{array}{c} {\;}^{W}T_{bl}^{∠}:\;\|{\text{J}}_{bl}\ddot{q}-{\ddot{x}}_{r}{\|}_{{\;}_{\text{W}}}^{2},\\ {\ddot{x}}_{r}={\overset{\cdot }{\omega }}_{d}-{\overset{\cdot }{J}}_{bl}\overset{\cdot }{q}-{\text{K}}_{P,o}{\text{e}}_{o}+{\text{K}}_{D,o}\left(\omega_{d}-\omega \right). \end{array} \end{array}$$

Where ***e*_*o*_** is the orientation error computed through quaternions^8^. The task is defined w.r.t. the world frame W while the superscript ∠ means that only the orientation part of the task is considered. In (8), $\omega_{d},\omega \in {\mathbb{R}}^{3}$ are the desired and measured angular velocity, respectively and ${\overset{\cdot }{\omega }}_{d}$ is the desired angular acceleration.

To track posture references, we define a postural task $T_{\ddot{q}}$:

(9) $$\begin{array}{l} \begin{array}{c} T_{\ddot{q}}:\;∥{\ddot{q}}_{a}-{\ddot{q}}_{r}{∥}_{W}^{2},\\ {\ddot{q}}_{r}={\text{K}}_{P,p}\left({\text{q}}_{a,d}-{\text{q}}_{a}\right)-{\text{K}}_{D,p}\left({\overset{\cdot }{q}}_{a,d}-{\overset{\cdot }{q}}_{a}\right), \end{array} \end{array}$$

defined just for the *actuated* part of (1). The posture references aim to keep the robot in a well-behaved kinematic configuration as in Figure 2. These references can be changed if the user wants to set a different height for the robot^9^. To ensure contact stability, it is common to constrain the contact forces to lie in a *linearized friction cone* $C_{fc_{i}}$ for each contact:

(10) $$\begin{array}{l} C_{fc_{i}}:\;\left\{ \begin{array}{c} F_{c_{i},\text{n}}\geq 0,\\ |{\text{F}}_{c_{i},\text{t}}|\leq \frac{\sqrt{2}}{2}\mu_{i}F_{c_{i},\text{n}}, \end{array}\right. \end{array}$$

where *F*_*c*_*i*_, n_ is the normal component, ${\text{F}}_{c_{i},\text{t}}\in {\mathbb{R}}^{2}$ are the tangential components of the contact force at foot *i* and μ_*i*_ is the friction coefficient. We also set some bounds on the contact forces:

(11) $$\begin{array}{l} C_{F_{c}}:={\underline{\text{F}}}_{c}\leq {\text{F}}_{c}\leq {\bar{\text{F}}}_{c}, \end{array}$$

and on the joint accelerations:

(12) $$\begin{array}{l} C_{\ddot{q}}:=\underline{\ddot{\text{q}}}\leq \ddot{\text{q}}\leq \bar{\ddot{q}}. \end{array}$$

Notice that the limits in (11) are chosen to be feasible upper and lower bounds w.r.t. the limits in (10).

### 2.2. Inequality Hierarchical Quadratic Programming (iHQP)

With all the *ingredients* presented before we set-up a cascade of constrained QP problems in the variables $\text{x}={\left[{\ddot{q}}^{T},\;{\text{F}}_{C}^{T}\right]}^{T}$:

(13) $$\begin{array}{l} \begin{array}{c} {\text{x}}_{\text{k}}^{*}=\underset{{\text{x}}_{\text{k}}}{\mathrm{argmin}}\;∥{\text{A}}_{\text{k}}{\text{x}}_{\text{k}}-{\text{b}}_{\text{k}}{∥}_{w}^{2}+\lambda ∥{\text{x}}_{\text{k}}{∥}^{2}\\ \text{subject to}\\ \;{\underline{\text{c}}}_{\text{k}}\leq \text{C}{\text{x}}_{\text{k}}\leq {\bar{c}}_{\text{k}}\\ \;{\underline{\text{u}}}_{\text{k}}\leq {\text{x}}_{\text{k}}\leq {\bar{u}}_{\text{k}}\\ \;{\text{A}}_{\text{j}}{\text{x}}_{\text{j}}^{*}={\text{A}}_{\text{j}}{\text{x}}_{\text{k}} \end{array} \end{array}$$

given a generic task **A**_k_**x** = **b**_k_ subject to the constraint **C**_k_ and bounds, for the *k*-th level of priority. The equality constraint enforces the priorities from all the previous *j* levels, with *j* = 0, …, *k* − 1. Notice that ${\text{x}}_{\text{j}}^{*}$ is the solution given by the previously solved QPs. The second term in the cost function is a regularization term for the *k*-th level through the λ gain. A regularization term on the ground reaction forces is mandatory to prevent ill-conditioning of the Hessian, avoiding instability in the solution.

In addition, the regularization of the contact forces can be used to prevent the solver from generating a solution with unnecessarily high forces or to increase robustness. For instance, in Focchi et al. (2017) regularization is used to find a solution where the forces try to be far away from the friction cone boundaries while in Nakamura et al. (1987) are inserted as a last priority task to minimize internal forces.

### 2.3. Stack of Tasks

The iHQP problem in (13) is used to solve two different *Stack of Tasks*, composed of the tasks and the constrains we introduced in the previous section. Despite the fact that the introduced whole-body control framework is generic w.r.t. the type of tasks and the number of priorities, we focus on only two kinds of stacks.

We first introduce the stack $S_{3}$ constituted by 3 levels of priorities:

(14) (∑i∈Ist WTci→/TW bl∠/Tq¨)≪Cfb≪Cfc≪Cq¨≪CFc

where the “/” symbol implies a null-space relation between the cost functions (*hard* hierarchy) and the “≪” symbol considers the insertion of constraints, in this case, to all the priority levels. Notice that we enforce contacts (*I*_*st*_ is the set of the indexes of the stance feet) as the first priority level, while in the secondary level we control the orientation of the base. Finally a postural task attracts the posture of the whole robot to a *nominal* reference. All these tasks are subject to be consistent with dynamics, friction cones, joint acceleration limits and force limits.

The second stack $S_{1}$ consists of a single level of priority constituted by a constrained weighted sum of tasks (*soft* hierarchy):

(15) S1:=(∑i∈Ist WTci→+TW bl∠+Tq¨)        ≪Cfb≪Cfc≪Cq¨≪CFc,

where the + operator is used to sum cost functions.

As a matter of fact, strict hierarchies in $S_{3}$ eliminate inconsistencies which may be generated by employing a single priority level, for example breaking contacts due to motion of the trunk. However, they can sometimes be too conservative so that they may completely block lower-priority tasks. On the other hand, classic implementation of strict priorities, as in $S_{3}$, rely on resolving a cascade of QPs which augment the computational cost w.r.t. a single priority level. Furthermore, up to a certain extent, it is possible to tune relative weights in $S_{1}$ so that the behavior will be similar, but not exactly the same, as in $S_{3}$ (if the ratio of the weights of different priority levels is sufficiently high, i.e., at least 10^3^)^10^. Therefore, $S_{1}$ is preferable for a real-time compatible implementations but, due to the presence of the weights, it becomes harder to fine-tune the different task contributions.

It is important to note that the presented approach does not rely on explicit control of the CoM of the robot. For stability purposes we instead rely on the postural task which acts in the final layer of $S_{3}$, or at low priority in $S_{1}$. The postural task will move the robot to a nominal configuration by exploiting the remaining Degrees of Freedom from the higher priority tasks, thus resulting in a motion that aligns the base with the stance feet. Avoiding direct control of the position of the CoM of the robot also has the advantage of achieving automatic adjustment of the base in the presence of uneven terrain, as will be shown later.

**Figure 3:**
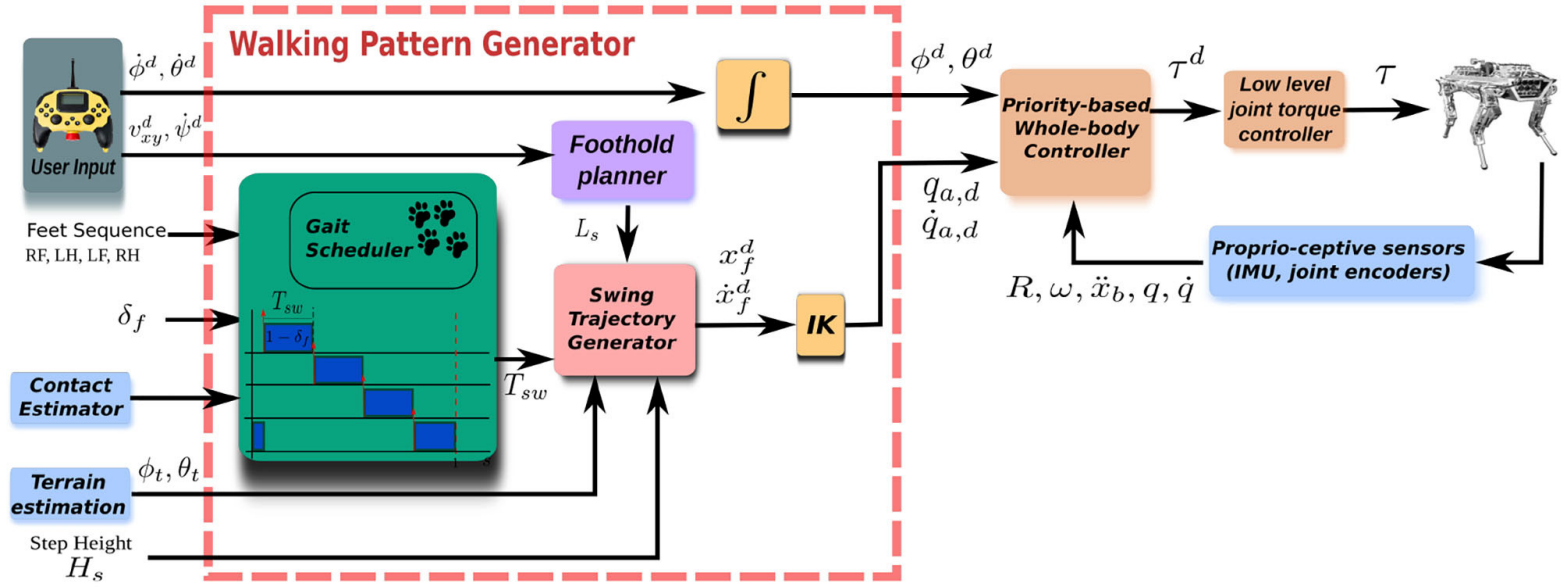
Block diagram of the locomotion framework. The user gives high-level velocity inputs to the foothold planner, that, on its behalf, is triggered by the gait scheduler. The swing trajectory generator computes the trajectories for the swinging feet, given the step length ${\Delta \bar{L}}_{xy}$ provided by the foothold planner for each swing leg.

The outputs of the QP, used to solve the problem described in (14) and (15), are optimal joint accelerations ${\ddot{q}}^{*}$ and contact forces ${\text{F}}_{c}^{*}$ that, plugged in (3), return the reference torques τ^*^. These will be the inputs of a low-level torque controller active at the joints of the robot. Figure 3 shows a block diagram of the components of the framework.

It is worth pointing out that the role of the inertia matrix **M**, which multiplies the joint accelerations ${\ddot{q}}^{*}$ (the controllers are written at the acceleration level), works as a time-varying, non-linear, and non-diagonal gain matrix acting on the feedback gains of the tasks, which are, most of the time, diagonal. This has the final effect of creating coupling between joints. A Cartesian task further increases the coupling because a Cartesian error is spread on several joints. This is an issue in the case where the tracking of a joint is worse than the others^11^. However, through a particular choice of the weight matrix and the feedback gains of the tasks it is possible to get back the diagonal gains achieving an equivalent Cartesian impedance controller (see Appendix A).

### 2.4. State Estimation and Contact Estimation

#### 2.4.1. Independence of Constraints From State Estimation

As stated in the previous section, to be robust w.r.t. state estimation drift, our intention is to drop the dependency from the *position* of the floating base w.r.t. the inertial frame. However, the orientation of the floating base and its angular velocity are still estimated using an IMU sensor. Neglecting the linear position (and velocity) of the base is analogous to continuously resetting the world frame origin to the base origin.

However, Equation (7) for the contact, is a constraint ${\text{J}}_{c}\ddot{q}=-{\overset{\cdot }{J}}_{c}\overset{\cdot }{q}$ that should be written in an inertial frame (not in the base frame), because the velocity of a foot depends not only on joint velocities but also on the motion of the floating base. Therefore, we are considering the floating base part in the **J**_*c*_ Jacobian. Apparently, it might seem that the linear part ***ẋ***_*b*_ of the base twist in $\overset{\cdot }{q}={\left[{\dot{x}}_{b}^{T}\;\omega^{T}\;{\overset{\cdot }{q}}_{j}^{T}\right]}^{T}$ is required (while we consider it to be zero).

However, if we carefully inspect the structure of ${\overset{\cdot }{J}}_{c}$, we notice that this matrix has columns of zeros multiplying the ***ẋ***_*b*_ variables, because **J**_*c*_ depends only on base orientation and on **q**_*j*_ but not on base linear position. This makes the term ${\overset{\cdot }{J}}_{c}\overset{\cdot }{q}$ dependent on ***ω*** but not on ***ẋ***_*b*_. Therefore considering ***ẋ***_*b*_ = **0** is not affecting the validity of Equation (7). The ***ẋ***_*b*_ seems to appear also in the dynamic Equation (3) (inside $\overset{\cdot }{q}$) that we enforce as equality constraint. However the term $\text{h}\left(\text{q},\overset{\cdot }{q}\right)$ is also independent from the base linear velocity making also this constraint unaffected. For the above reasons, the theoretical foundation of our approach is still perfectly valid even if we consider both **x**_*b*_, ***ẋ***_*b*_ = **0**, making our locomotion independent from the need of a state-estimation algorithm.

#### 2.4.2. Floating Base Height Estimation

To be able to control the height of the robot it is necessary to obtain an estimation of it. Differently from the *X* and *Y* coordinates, the base height can be considered as the average *relative* position of the feet in the H frame, which can reliably be estimated through the forward kinematics of the feet ${\;}^{B}{\text{x}}_{c_{i}}$.

(16) xH ci=RH BxB ci,

(17) xH bl,z=−1N∑i∈Ist Hxci,z

where the rotation matrix ${\;}^{H}{\text{R}}_{B}$ encodes the orientation of the base w.r.t. the H frame which can be easily measured with the IMU, and *I*_*st*_ is the set of the indexes of the stance feet and *N* their number.

#### 2.4.3. Contact Estimation

In order to understand which are the active contacts, we rely on contact force estimation based on torque readings extracting the leg equation from (3)^12^:

(18) $$\begin{array}{l} {\text{F}}_{c_{i}}=-{\text{J}}_{c_{i}}^{-T}\left(\tau_{c_{i}}-{\text{h}}_{i}\right), \end{array}$$

where ${\text{F}}_{c_{i}}\in {\mathbb{R}}^{3}$ is the estimated contact force of one leg, ${\text{J}}_{c_{i}}\in {\mathbb{R}}^{3\times 3}$ is the leg Jacobian and ***τ***_*c*_*i*__ are the measured torques in one leg and ${\text{h}}_{i}\in {\mathbb{R}}^{3}$ is the Coriolis/Centrifugal and gravity bias. When the projection of **F**_*c*_*i*__ along the normal to the terrain overcomes a certain threshold (Focchi et al., 2020), we consider the leg to be in contact with the environment.

## 3. Walking Pattern Generator

The walking pattern generator receives desired twist commands for the base of the robot from an external source, such as an operator device or a high level planner^13^, and transforms these into swing trajectories for the legs given a specific type of gait. In order to do so, the walking pattern generator is composed by (A) a gait scheduler that sequences the footsteps based on the gait, (B) a foothold planner which transforms the desired base twist commands into footholds by using the horizontal frame H as reference frame, (C) a swing trajectory generator which takes the foothold coordinate and the desired step height as inputs, and calculates the swing trajectory, (D) an inverse kinematics transformation to map the leg's trajectories from Cartesian to joint space. Note that we decided to implement the swing trajectory in the joint space rather than in the Cartesian space because of the coupling issues described in the previous section.

### 3.1. Gait Scheduler

**Figure 4:**
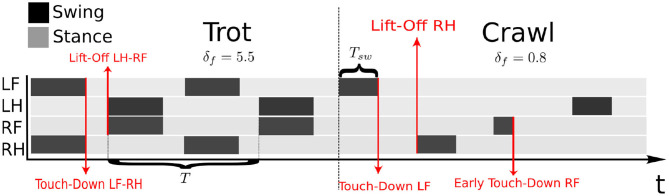
The left side of the image shows the schedule of a trot gait, while the right side shows a crawl gait. Given the feet sequence, the scheduler triggers the lift-off events for the legs. The touch-down events instead, are triggered if a real contact is detected (*haptic touchdown*) by the contact estimator. The time taken to complete a step cycle is defined as *T*. The ratio between the stance duration and the cycle duration is defined as the duty factor parameter δ_*f*_ = *T*_*st*_/*T*. Consequently, the swing duration is computed as *T*_*sw*_ = *T*(1 − δ_*f*_) and the swing frequency as *f*_*sw*_ = 1/*T*_*sw*_. In this example, the trot has a δ_*f*_ = 0.55 while the crawl has δ_*f*_ = 0.8, therefore, the crawl keeps the feet for a longer time on the ground. Indeed, a high value for δ_*f*_ can be useful for slower gaits, such as the crawl to give time to the whole-body controller to recover the posture. Note that during the stance the contacts of the legs are enforced by the whole-body controller. The scheduler can trigger the swing only if the corresponding leg has completed a step cycle.

Each different gait can be simply defined as a timed sequence of footsteps. Therefore, given a type of gait, the role of the scheduler is to trigger the sequence of leg swings (see Figure 4).

**Figure 5:**
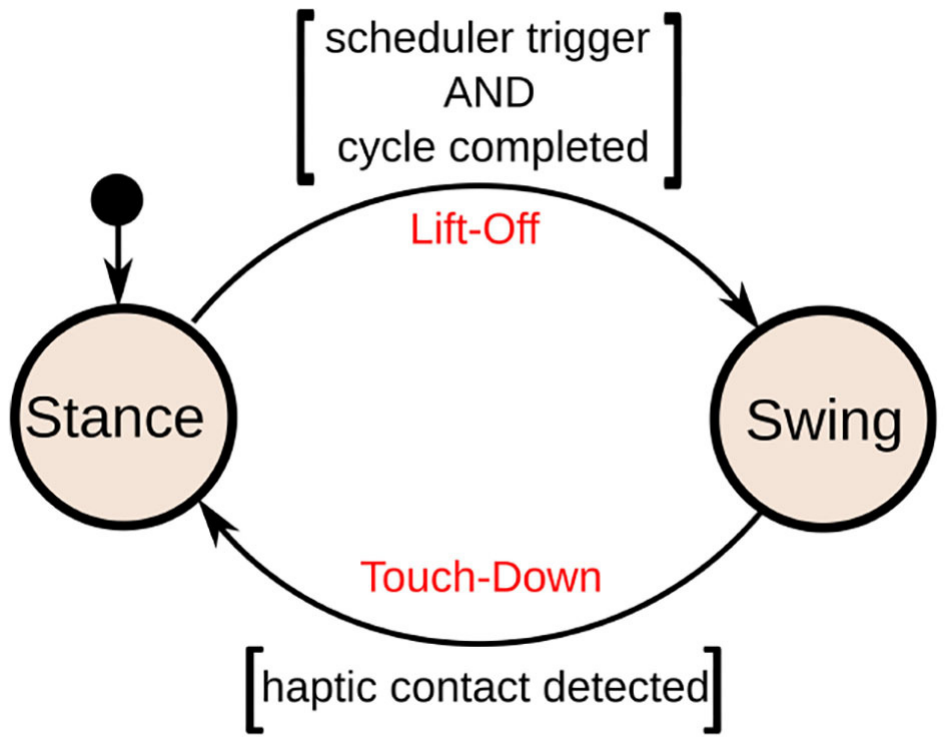
Each leg starts in the **Stance** state, waiting for the scheduler to trigger the swing. When triggered, the state machine switches from **Stance** to **Swing**. The state machine switches from **Swing** to **Stance**, if a contact is detected by the contact estimator. The step cycle duration *T* determines the time the leg has to be in the **Stance** state, before it can be triggered by the scheduler for a new swing.

A state machine is associated to each leg to keep track of its state. The possible states and transitions are depicted in Figure 5.

### 3.2. Foothold Planner

**Figure 6:**
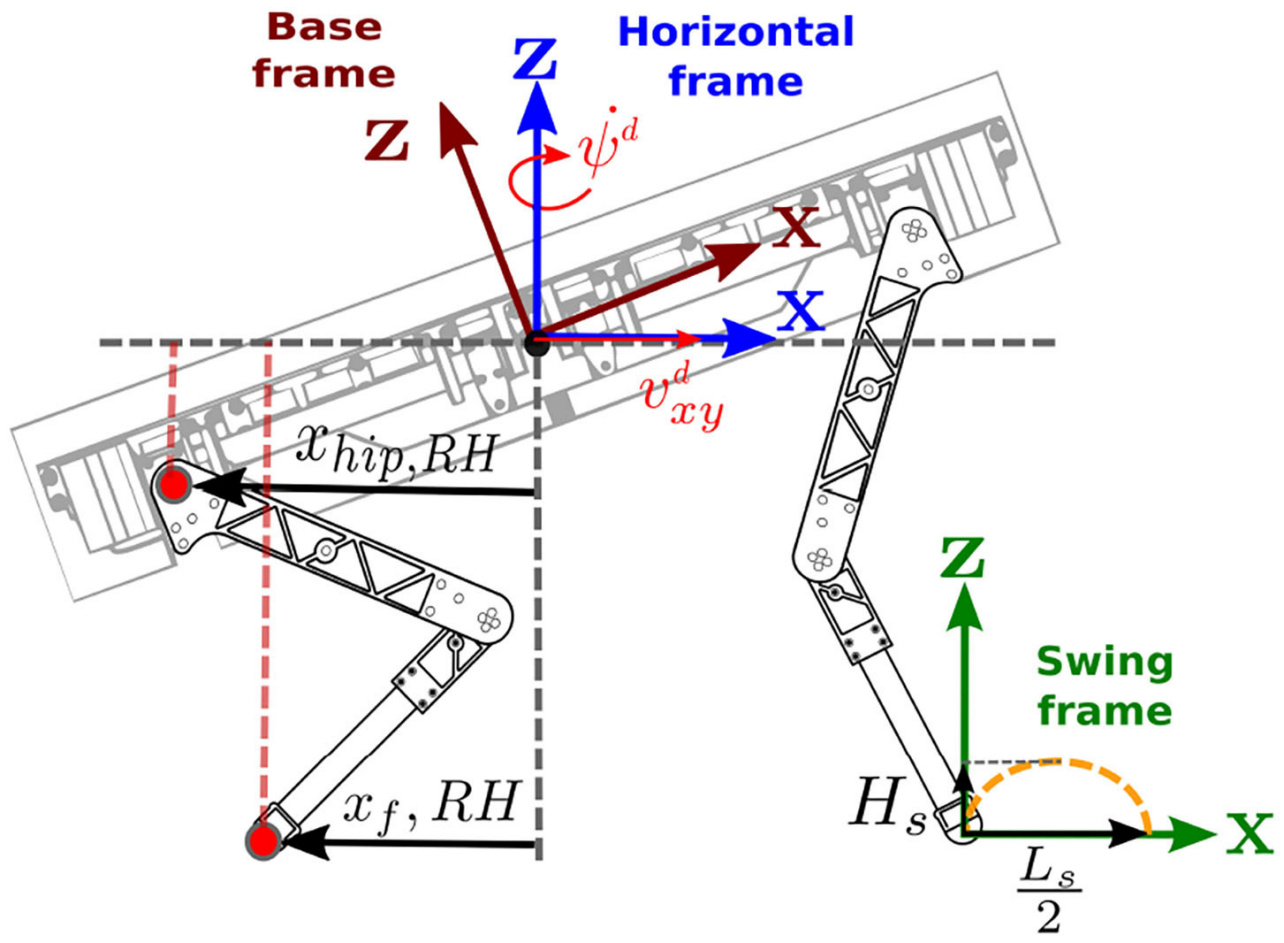
Frames used by the foothold planner. Desired base twist and foothold positions are expressed in the horizontal frame. Each step is taken with respect to a “virtual foothold” that moves with the base and represents the nominal size of the stance of the gait cycle (e.g., when the desired twist is set to zero).

**Figure 7:**
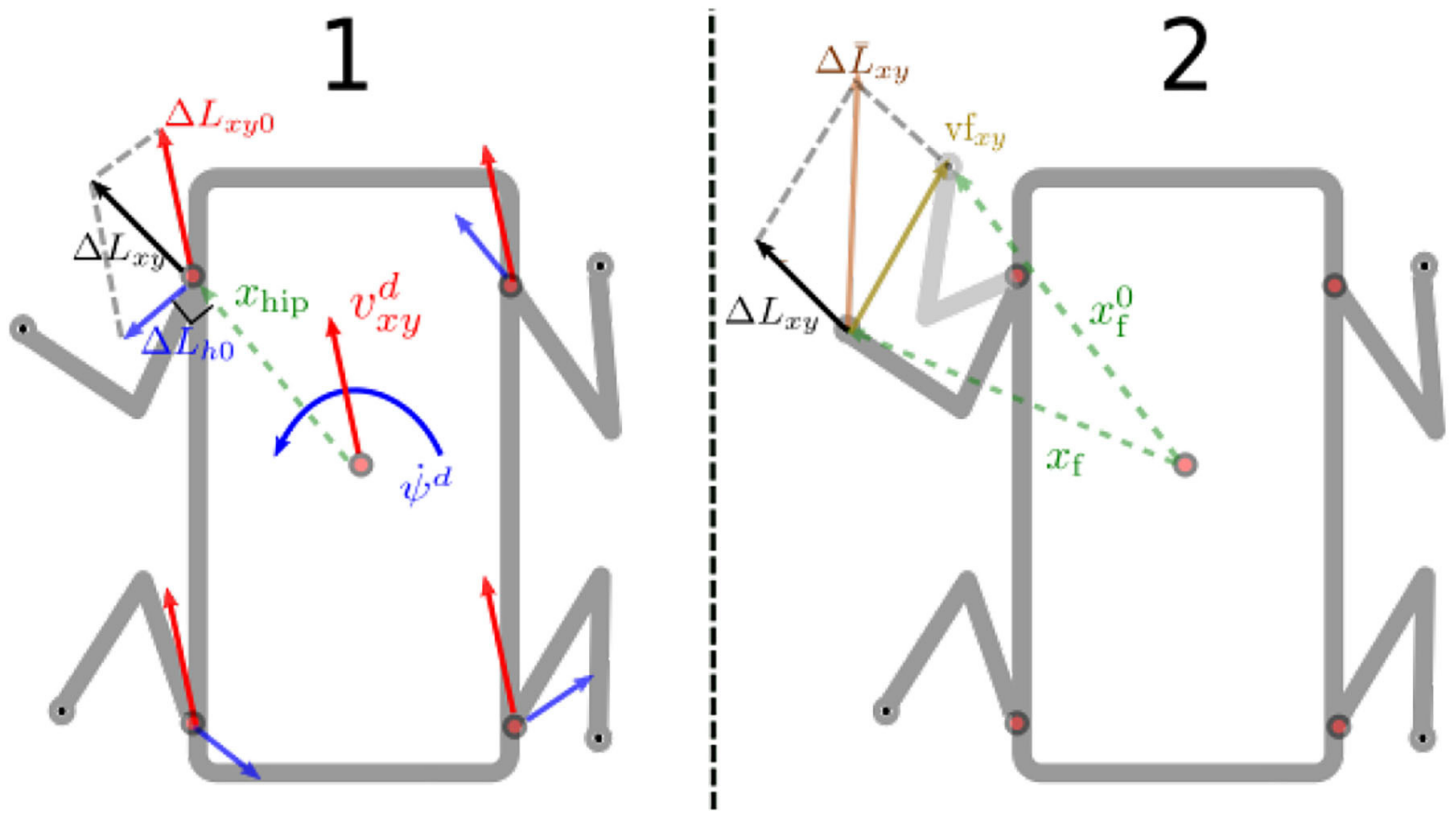
Top view of the robot with the geometrical explanation of the foot displacements computation: (1) starting from the desired linear ${\text{v}}_{xy}^{d}$ and angular $\overset{\cdot }{\psi^{d}}$ velocities, we compute the corresponding deltas **ΔL**_*xy*0_ and **ΔL**_*h*0_. (2) The resulting vector **ΔL**_*xy*_ is then summed to the virtual foothold vector **vf**_*xy*_ to produce the total foot displacement $\Delta {\bar{L}}_{xy}$.

The foothold planner calculates the desired foothold coordinates (*X* and *Y*) in the H frame (see Figure 6). Choosing such reference frame for the foothold selection makes the swing trajectory generation *independent* from the roll and pitch orientation of the base. Henceforth, unless specified, we assume all the vectors are expressed in that frame. The foothold coordinates are computed starting from the *desired* linear $v_{x}^{d}v_{y}^{d}$ and yaw angular velocity $\overset{\cdot }{\psi^{d}}$ of the base. These two velocities are transformed into foot displacements $\Delta {\text{L}}_{xy0}\in {\mathbb{R}}^{2}$ (see Figure 7 and Equations 19, 20). These deltas are not added to the previous foot position but to a *virtual foothold* offset that is computed with respect to the *actual* position of the base. This “robo-centric” foothold selection is an important feature to increase the robustness when dealing with rough terrain because it avoids accumulation of errors that would appear if the steps are taken w.r.t. the previous foot positions and allows to keep the robot close to a preferred kinematic configuration (the absence of this mechanism would make the robot legs stretch or compress).

The linear part of mapping of the desired velocity command is:

(19) $$\begin{array}{l} \Delta {\text{L}}_{xy0}=\frac{1}{f_{sw}}\left[\begin{array}{c} v_{x}^{d}\\ v_{y}^{d}\\ 0 \end{array}\right], \end{array}$$

which represents the displacement of the foot produced by a linear velocity command, *f*_*sw*_ is the step frequency. For the angular part instead, we have the following:

(20) $$\begin{array}{l} \Delta {\text{L}}_{h0}=\frac{1}{f_{sw}}\left[\begin{array}{c} 0\\ 0\\ \overset{\cdot }{\psi^{d}} \end{array}\right]\times {\text{x}}_{\text{hip}}, \end{array}$$

where **x**_hip_ represents the position of the hip of the swinging leg, in the horizontal frame. By summing these two quantities we obtain **ΔL**_*xy*_:

(21) $$\begin{array}{l} \Delta {\text{L}}_{xy}=\Delta {\text{L}}_{h0}+\Delta {\text{L}}_{xy0} \end{array}$$

To keep the robot close to a preferred stance configuration, and avoid accumulation of errors, we compute the difference between a preferred *virtual foothold* location defined as ${\text{x}}_{\text{f}}^{0}$ and the current one **x**_f_. Therefore, we obtain the following offset:

(22) $$\begin{array}{l} {\text{v}\text{f}}_{xy}={\text{x}}_{f}^{0}-{\text{x}}_{f}, \end{array}$$

which is then summed to (21) to obtain the total foot displacement:

(23) $$\begin{array}{l} \Delta {\bar{L}}_{xy}=\Delta {\text{L}}_{xy}+{\text{v}\text{f}}_{xy}. \end{array}$$

Note that in absence of base command velocities, the effect of the offset (22) is to align the feet to the virtual foothold configuration.

Finally we extract from $\Delta {\bar{L}}_{xy}$ the step length and *L*_*s*_ the angle ψ_*s*_ of the swing trajectory plane as follows:

(24) $$\begin{array}{l} L_{s}=\sqrt{{\Delta {\bar{L}}_{x}}^{2}+{\Delta {\bar{L}}_{y}}^{2}} \end{array}$$

(25) $$\begin{array}{l} \psi_{s}=atan2\left(\Delta {\bar{L}}_{y},\Delta {\bar{L}}_{x}\right) \end{array}$$

### 3.3. Swing Trajectory Generator

The swing is generated in the swing frame S (see Figure 6 for frame definitions) and is defined as an ellipse built up by mean of sine functions. This has the advantage to easily create a reaching motion and re-plan the trajectory if the user decides to change the swing duration (e.g., by changing the duty cycle or the cycle duration). The swing frame is the same as the horizontal frame (e.g., shares the same yaw orientation) but it is aligned with the terrain and has the origin in the swinging foot. We assume that a terrain estimator is providing the local inclination of the terrain (Focchi et al., 2020) in roll ϕ_*t*_ and pitch θ_*t*_. Thanks to the continuous re-computation of the step length *L*_*s*_ and the step heading ψ_*s*_, it is also possible to constantly re-plan the trajectory based on the desired base twist and current base configuration^14^ as explained in the previous section 3.2.

The trajectory for the swing (expressed in the swing frame S) is computed as follows:

(26) $$\begin{array}{l} {\;}^{S}x_{f,x}=\frac{L_{s}}{2}\left(1-cos\left(\pi f_{sw}t\right)\right),\\ {\;}^{S}x_{f,y}=0,\\ {\;}^{S}x_{f,z}=H_{s}sin\left(\pi f_{sw}t\right), \end{array}$$

where *L*_*s*_ and *H*_*s*_ are respectively the step length and the step height, and *t* ∈ [0, *T*_*sw*_]. While the step length *L*_*s*_ is defined by the foothold planner, the step height can be arbitrarily chosen based on the presence of obstacles or the type of terrain. After mapping (26) to the inertial frame, we obtain:

(27) xW f=RW S xS f,

where ${\;}^{W}{\text{R}}_{S}$ maps vectors from the swing to the inertial frame, and it is defined as a rotation of ψ_*s*_ + ψ^15^ along the *Z* axis and a rotation about the *X* an *Y* axes of ϕ_*t*_ and θ_*t*_ (if an estimation of the terrain inclination is available). Since the trajectory for the velocity is defined in a moving frame, its derivative must be computed taking the chain rule derivatives of (27) and (26):

(28) x˙W f=R˙W S xS f+ RW S x˙S f,

with ${\;}^{S}{\dot{x}}_{f}$ defined as:

(29) $$\begin{array}{l} {\;}^{S}{\dot{x}}_{f,x}=\pi f_{sw}\frac{L_{s}}{2}sin\left(\pi f_{sw}t\right),\\ {\;}^{S}{\dot{x}}_{f,y}=0,\\ {\;}^{S}{\dot{x}}_{f,z}=\pi f_{sw}H_{s}cos\left(\pi f_{sw}t\right). \end{array}$$

While the timing of the lift-off is dictated by the scheduler, the touch-down is triggered haptically (Focchi et al., 2020) to be sure that the stance is only triggered when a stable foothold is established. During the swing down phase, the occurrence of the contact with the terrain is continuously checked. The swing can continue beyond the planned foothold (reaching motion) until the contact is detected when *t* ≥ *T*_*sw*_. The foot is considered in contact with the ground when the ground reaction force overcomes a certain threshold in the direction normal to the terrain. The *haptic* touch-down is a crucial feature to address rough terrain because it prevents to inject destabilizing forces to the base created by the tracking of trajectories that are not terrain consistent.

### 3.4. Inverse Kinematics

To transform the swing trajectories from Cartesian to joint space we use the Closed Loop Inverse Kinematics (CLIK) algorithm (Siciliano et al., 2009). Therefore, for each swinging leg *i* we can express the joint velocity as:

(30) q˙ai,d=Jci−1[x˙W fi,d+P(xW fi,d− xW fi)],

where **P** ≥ 0 is the CLIK proportional gain, ${\;}^{W}{\text{x}}_{f_{i}}$ represents the current foot position w.r.t. the inertial frame, ${\;}^{W}{\text{x}}_{f_{i},d}$ and ${\;}^{W}{\dot{x}}_{f_{i},d}$ are the desired velocity and position reference provided by the swing trajectory generator through the Equations (27) and (28), respectively. *q*_*a*_*i*_, *d*_ is found by integration. In order to control the base height, it is possible to reconfigure the legs in stance. For example, in order to increase the height of the base, the robot's legs must be stretched, while to decrease it, the legs must be retracted. Therefore, we can map the desired base height to the joint positions for the legs *i* in stance as:

(31) $$\begin{array}{l} \begin{array}{c} {\overset{\cdot }{q}}_{a_{i},d}={\text{J}}_{c_{i}}^{-1}\;\text{P}{\;}^{W}\Delta {\text{h}}_{bl}, \end{array} \end{array}$$

where ${\;}^{W}\Delta {\text{H}}_{bl}=\left[0,0,{-}^{W}\Delta H_{bl,z}\right]$ is a vector that defines the desired change of height for the base of the robot. The value ${\;}^{W}\Delta H_{bl,z}$ is defined as:

(32) $$\begin{array}{l} {\;}^{W}\Delta H_{bl,z}{=}^{W}x_{bl,z,d}{-}^{W}x_{bl,z}, \end{array}$$

where ${\;}^{W}x_{bl,z,d}$ represents the desired base height and ${\;}^{W}x_{bl,z}$ is the actual base height estimated with (17) as described in section 2.4.2^16^. Finally, to track the joint space references both for the feet in stance and in swing, we use the postural task $T_{\ddot{q}}$ introduced in (9).

## 4. Implementation Details

In this section we present some implementation details and remarks on the final control scheme that we are employing on the real platform.

### 4.1. Swing Task

The swing task can be implemented as a Cartesian task or a joint task. From a theoretical point of view, a Cartesian space formulation is more sound because it allows us to set the gains in the same space the trajectories are defined. Conversely, a joint space formulation provides an anisotropic and tilted impedance ellipsoid at the feet, making the legs more compliant in a direction than in another depending on the leg configuration, even with a constant joint stiffness. However, in the implementation on the real HyQ platform, we found issues with the Cartesian implementation of the swing task. The reason is that the Jacobian matrix *couples* the tracking errors of all the joints. This is not a problem if they are all able to perform a good tracking. However, with our platform HyQ, the distal limbs of the legs (lower-legs links) are very light and their load-cells measure barely zero-torque during the swing^17^ and consequently, the feed-back loop opens. Therefore, the only way to make the joint move, is to create a position error that is big enough to increase the desired torque even if the actual torque remains zero. When implementing Cartesian impedance control algorithms for the swing legs, this peculiarity of the lower leg joint affects performance as well the other joints in the leg. Conversely, with the joint space implementation we are able to avoid this coupling between the joints. For this reason, we have chosen a joint space implementation and the swing references are sent directly to the postural task. However, with this implementation, the coupling due to the inertia matrix is still present, and the matrices **K**_*P*_, and **K**_*D*_ assume the meaning of acceleration gains rather than joint impedance gains. To avoid this problem, it is possible to pre-multiply these gains with the inverse of the inertia matrix, giving them the physical meaning of joint *stiffness* and *damping* (see Appendix A). This formulation turns out to be beneficial to improve the tracking of the swing legs.

### 4.2. Force and Acceleration Bounds

To handle contact transitions, during stance and swing phases, we impose contact force constraints to switch between a maximum allowed value and zero. This allows to keep the size of the QP problem constant during the whole locomotion phase, which can be useful in a hard real-time implementation. To prevent torque discontinuities it is possible to implement a smooth unloading/loading by setting a time-varying upper bound on the contact force as in Focchi et al. (2017). During preliminary experiments performed on the real robot, we found that there is a strong influence between the acceleration bounds and the tracking accuracy. In particular, setting the limits too low results in an *overshoot* with the tracking of the desired trajectory at the touchdown (when there is the biggest deceleration). This problem appears only on the real robot and is not present in simulation, because in this second case, the tracking errors are smaller.

The acceleration and the force limits (active only during the stance phase), are summarized in Table 2 together with the other parameters set in the controller.

**Table 2:** Parameters used in the controller.

Hessian regularization factor	η	1*e* − 6
Force Max X	${\bar{f}}_{x}$	1,000 [N]
Force Max Y	${\bar{f}}_{y}$	1,000 [N]
Force Max Z	${\bar{f}}_{z}$	1,000 [N]
Force Min X	${\bar{f}}_{x}$	1,000 [N]
Force Min Y	${\bar{f}}_{y}$	1,000 [N]
Force Min Z	${\bar{f}}_{z}$	20 [N]
Accel Max	${\ddot{q}}_{max}$	500 [*rad*/*s*^2^]
CLIK gain	*P*	10 [1/*s*]
Swing frequency	*T*_*sw*_	2 [*Hz*]
Step height	*H*_*s*_	0.1 [*m*]

### 4.3. Haptic Touch-Down Event

To keep spurious contact estimations from triggering a premature touch-down in the leg's state machine, it is possible to disable the haptic contact detection during the swing up phase (half of the swing time). To detect the touchdown the threshold on the ground reaction forces is set to 50 N (see section 2.4).

### 4.4. Loop Frequency

The output of the whole-body controller is given as a desired torque to the low-level torque controller, in a cascade loop architecture. Both the whole-body controller and the low-level torque controller run at 1 *kHz*. In the implementation on the real robot, the trunk controller damping is limited to a max of 400 *Nmd*/*rad* to avoid instabilities, because the loop frequency is known to limit the maximum value for the damping (Focchi et al., 2016).

### 4.5. Solver and Computation Time

To solve the stack *S*_3_, we used the solver qpOASES (Ferreau et al., 2014), leveraging on the whole-body control framework OpenSoT (Mingo Hoffman et al., 2017). With an Intel Quad-Core i5-4440 CPU @ 3.10 GHz (onboard) machine, it requires on average 1,180 ± 20 μ*s* to solve the three layers. Conversely, with the single stack *S*_1_ the computation time drops to 830 ± 20 μ*s*, making this implementation preferable to be run at 1 kHz. It is worth noting that most of the optimization time is spent in calculating the Hessian.

### 4.6. Terrain Estimator

If a terrain estimation algorithm (Focchi et al., 2020) is available, a reference can be given to the orientation task to align the base with the slope of the ground and prevent reaching the kinematic limits of the leg. However, in the absence of a terrain estimator, the base orientation task can be removed from the stack and the postural task can be used to achieve some sort of terrain adaption, because it will attempt to align the base with the feet.

## 5. Experiments

**Figure 8:**
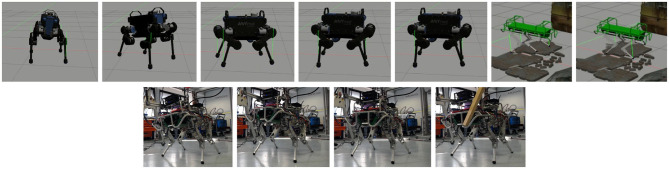
First row: snapshots of the locomotion simulations: ANYmal tracking a **(A)** roll reference, a **(B)** pitch reference, **(C,D)** changing the robot height. HyQ during a **(E)** crawl swinging only one leg at a time and a trot **(F)** swinging two legs at the time. Second row: snapshots of the trunk orientation experiments with HyQ. The robot is tracking an orientation reference while being disturbed by an external interaction.

In this section we present some experiments to demonstrate the effectiveness of our whole-body framework for quadruped robots (see the accompanying Supplementary Video 1^18^ and Figure 8 for a summary of all the experiments). The simulations have been carried out with the Robot Operating System (ROS) in a Gazebo environment^19^ that uses the ODE physics engine (Chitta et al., 2017). A friction coefficient of μ = 0.8 was set (unless specified) in all the experiments.

**Figure 9:**
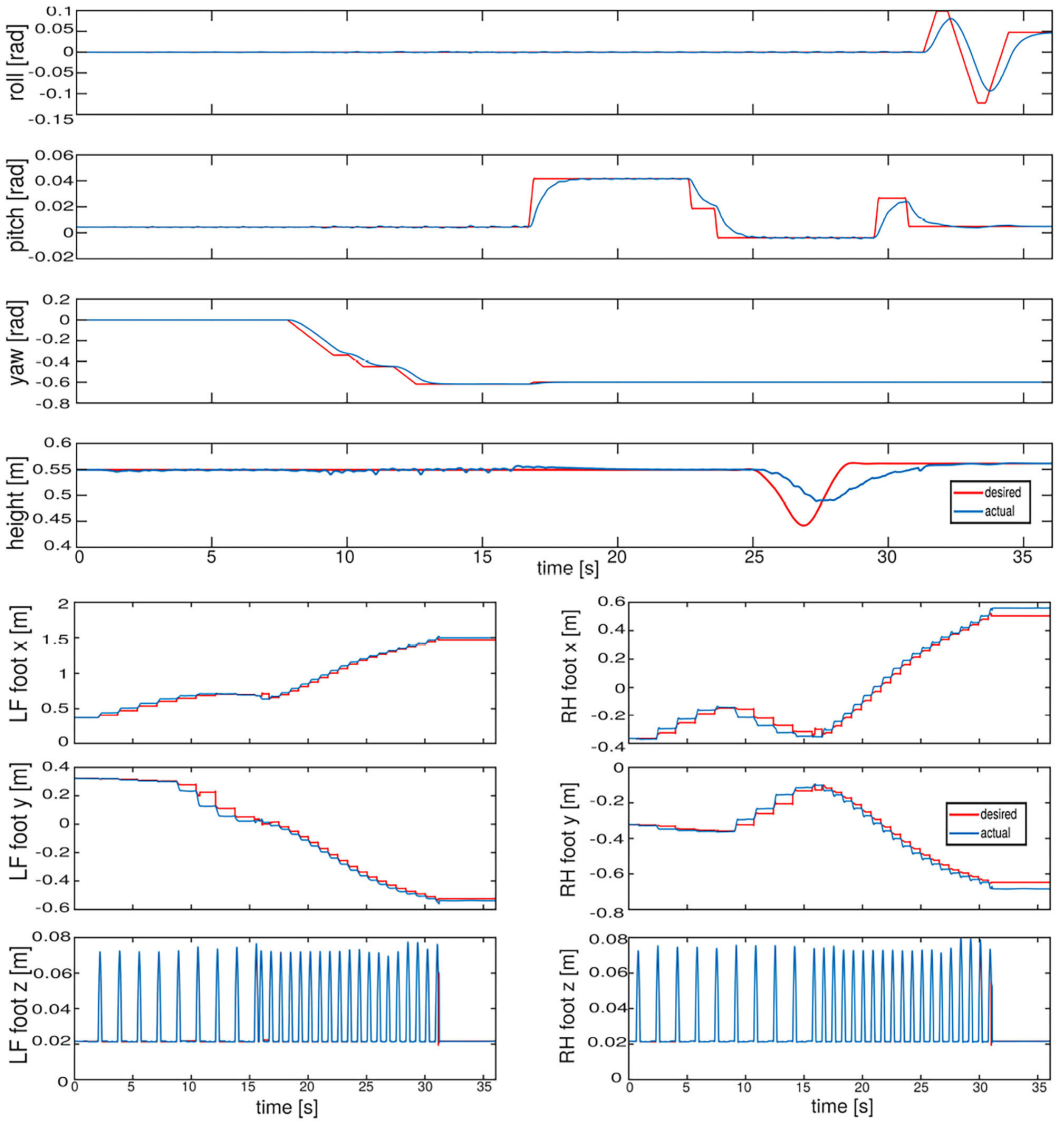
Simulation—HyQ walking on flat terrain: The upper plots show the tracking of the base orientation (roll, pitch, and yaw) and of the base height. Note that the height does not attain its reference, because it is implemented via the postural task that is in the null-space of the orientation task. In the lower plots instead, the tracking of the left front and right hind foot in the *X*, *Y*, and *Z* coordinates is shown. After ~15 s, the gait is switched from a crawl to a trot.

We tested our approach on two different quadruped platforms (HyQ and ANYmal) of different sizes and weights. The porting to a different platform required only a slight tuning of the gains of the postural and of the trunk orientation tasks. In a first simulation performed with HyQ we show in the Supplementary Video 1 that the robot is able to seamlessly switch between a crawl and trot. The robot is traversing a rough terrain area made of ruins and cobble-stones, moving omni-directionally. Notice that the robot is *blind* and not aware of the status of the terrain. To demonstrate the motion decoupling capability of our framework, the robot performs a walk on flat terrain while changing the base orientation and the height. Figure 9 shows the tracking of the base orientation and of the height in the upper plots, while in the lower plots is reported the tracking in Cartesian space for the LF and RH feet. The gains used for the Cartesian and postural tasks are reported below. For the base orientation (8) we set **K**_*P*_ = *diag*([1000.0, 1000.0, 1000.0]) and **K**_*D*_ = *diag*([100.0, 100.0, 100.0]). For the postural task (9) the gains are scheduled depending on the walking phase: for the *swing* phase we set **K**_*P*_*sw*__ = *diag*([300.0, 300.0, 300.0]), **K**_*D*_*sw*__ = *diag*([8.0, 12.0, 5.0]), while for the *stance* phase **K**_*P*_*st*__ = *diag*([500.0, 500.0, 500.0]), **K**_*D*_*st*__ = *diag*([20.0, 20.0, 20.0]). To improve tracking for the swing phase it is possible to pre-multiply the gains for the inverse of the inertia matrix (of the leg) (see Appendix A).

The ground truth coming from Gazebo is used to obtain the measurements in the world frame. In both cases, good tracking without steady errors is achieved; indeed the swing tasks and the base orientation task are not conflicting with each other because the latter is written in the horizontal frame which is independent from the base orientation. For completeness we present the mean and the standard deviation of the tracking errors during the whole experiment, in Table 3.

**Table 3:** Mean and standard deviation of the errors.

**Measurements**	**Mean**	**Std**
Roll	0.0050 [rad]	0.0145 [rad]
Pitch	0.0072 [rad]	0.0187 [rad]
Yaw	0.0017 [rad]	0.0043 [rad]
Height	0.0071 [m]	0.0145 [m]
RH–X	0.0303 [m]	0.0186 [m]
RH–Y	0.0203 [m]	0.0128 [m]
RH–Z	0.0016 [m]	0.0027 [m]
LF–X	0.0235 [m]	0.0152 [m]
LF–Y	0.0202 [m]	0.0201 [m]
LF–Z	0.0006 [m]	0.0022 [m]

**Figure 10:**
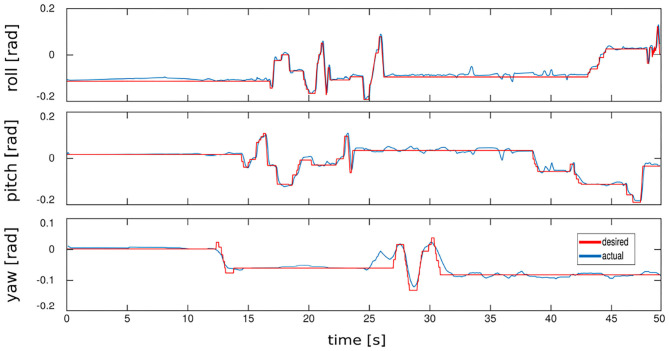
Real hardware—HyQ changing the base orientation in roll, pitch, and yaw.

We carried out preliminary experiments on the real platform HyQ showing a 2 Hz trot on flat terrain, in a second moment we control the base orientation to follow some operator desired reference commands given by mean of a joy-pad interface while an external disturbance acts on the robot (see Supplementary Video 1). The tracking error has an average of 0.0101 *rad* with a standard deviation of 0.0102 *rad* (Figure 10).

Remarks: To achieve a successful implementation on the real robot we had to do some modification on the original formulation of the optimization problem, see section 4.

## 6. Discussion

***Stance Task***: the first thing to notice is that we rely on the minimization of the Cartesian acceleration, to keep the feet in contact with the ground. The contact task is set at the highest level and consists of maintaining zero acceleration of the foot relative to the inertial frame. If, for some reasons, the contact is lost (e.g., due to uncertainties in estimating the terrain's normal direction or the friction coefficient) the feet would diverge because there is no feedback to keep the position unchanged (we chose to do this to make the formulation independent from the state estimation). This can lead to considerable motions of the stance feet with possible loss of stability. In a previous work (Fahmi et al., 2019) we implemented a specific task to keep the relative position of the feet in stance constant avoiding divergence. However, we noticed that the presence of the postural task that is acting in the null-space, naturally makes it up for this feature.

***Robustness***: as anticipated in the introduction, removing completely the CoM task does not give guarantees on locomotion stability. When one leg starts to swing it can happen that the opposite leg gets unloaded if the ZMP happens to be on the boundary of the support triangle. In this case the robot will start to tip over because the controller loses control authority. This is not a big issue if the swing frequency is high enough, because the stance can be quickly recovered, however it can become problematic for lower stepping frequencies. One way to mitigate this is to gradually unload the swing, by gradually reducing the upper bound on the contact force of the leg to swing, while keeping a minimum (non-zero) lower bound on the opposite leg. This way the optimization will naturally drive the CoM away from the boundary of the triangle in order to keep some residual “loading” on the leg opposite to the swing one, giving some robustness margin.

## 7. Conclusions

In this work we present a novel locomotion framework for quadrupedal robots that merges a walking pattern generator, acting only at the foot level, with a prioritized whole-body inverse dynamics controller. One of the advantages of the proposed framework is to avoid estimating the linear position and velocity of the floating base, while maintaining the ability to effectively tackle moderately rough terrain. This has been achieved by leveraging the postural task acting in the whole-body controller as a sort of elastic element. Consequently, the robot's base follows the feet, resulting in a motion of the trunk that adapts naturally to the foot stance configuration while trying to keep a well-behaved kinematic configuration. To increase the robustness of the proposed approach, the foothold selection is done w.r.t. a *virtual foothold* defined in the *horizontal frame* of the robot making the footstep strategy independent from the base orientation. In this way, no CoM planning is required to implement various types of gaits. However, despite the fact that presented framework is capable to handle uneven terrain, it relies on a particular posture which in turn may need to be properly tuned according to the particular type of terrain being traversed. As part of future work, we plan to further extend the proposed approach by taking into account the presence of a manipulator mounted on the robot's trunk. This would allow operation with complex loco-manipulation tasks. Since our approach is based on mixed *hard-* and *soft-*priorities, we will consider using machine learning techniques in order to properly find optimal weights between the different tasks.

## Data Availability Statement

The datasets generated for this study are available on request to the corresponding author.

## Ethics Statement

Written informed consent was obtained from the individual(s) for the publication of any potentially identifiable images or data included in this article.

## Author Contributions

The Scientific contribution is equally distributed among GR, EM, and MF (33% each). CS and NT contributed with funding, lab space and provided the robot platform for experiments. All authors contributed to the article and approved the submitted version.

## Conflict of Interest

The authors declare that the research was conducted in the absence of any commercial or financial relationships that could be construed as a potential conflict of interest.

## Footnotes

^1^Here the term “stability” is intended in the sense defined by Pang and Trinkle (2000).

^2^Priorities are achieved by projecting low priority task Jacobians by means of linear operators (i.e., the null-space projector of higher priority task Jacobians).

^3^The cost functions often quantify the error between a desired and a measured value and present it in the form of a squared euclidean norm.

^4^In particular we use Euler angles to represent the orientation of the base, so *f* = 6. Note that Euler angles can have singularity at 90° pitch, which is an orientation we never consider in our tasks.

^5^We assume our robot establishes *point* contacts with the environment.

^6^Note that, in general, the size of the weight matrix **W** depends on the size (number of *rows*) of the task.

^7^Even in the case the CoM position is estimated via *relative* measurements (based solely on relative encoders readings that are quite accurate), the occurrence of slippage in the foot chosen as reference, can make the estimation suffer from drift.

^8^The orientation error can be computed from the quaternion error ***α*_*e*_** = η_*e*_, ***ϵ*_*e*_** as ${\text{e}}_{o}=2arccos\left(\eta_{e}\right)\frac{\epsilon_{e}}{∥\epsilon_{e}∥}$. The quaternion error is computed from a desired orientation quaternion ***α***_*d*_ = [η_*d*_, ***ϵ*_*d*_**] and the actual orientation quaternion ***α*_*a*_** = [η_*a*_, ***ϵ*_*a*_**] as in Yuan (1988), $\alpha_{e}=\eta_{a}\eta_{d}+{\epsilon_{a}}^{T}\epsilon_{d},\eta_{a}\epsilon_{d}-\eta_{d}\epsilon_{a}-\epsilon_{a}\times \epsilon_{d}$.

^9^Note that this configuration works well for flat or moderate terrain inclinations and might pose stability issues for bigger inclinations; to improve robustness, it would be possible to make the robot lean forward when climbing up a ramp, as presented in Focchi et al. (2020). Improving the postural task in this regard is part of future work.

^10^This is a rule of “thumb” that works well in practice considering the tasks' normalized costs in SI units.

^11^In the case of the knee joint of HyQ, due to the low inertia of the link, the torque sensor barely measures any torque during the swing (when there is no contact), resulting in a open feedback loop. For further details see section 4.

^12^For the sake of simplicity, we discard the acceleration terms because their influence on the force computation is very low. However, in case the acceleration terms can not be neglected, they can be incorporated in the equation after filtering. The filtering is necessary due the presence of quantization noise, which would otherwise be amplified by the double differentiation of the encoder measurements.

^13^In our experiments we used a joy-pad connected to the operator's pc.

^14^Assuming smooth input changes.

^15^We remember that we computed ψ_*s*_ w.r.t. the H frame so we need to consider also the orientation ψ of this frame w.r.t. the W frame.

^16^Both values can be expressed either in the world or horizontal frame since the two differ only for a rotation around the *Z* axis.

^17^Load-cells and torque sensors in legged robots are sized in order to be able to measure big torques during the stance, for this reason they lack accuracy during the swing.

^18^Experiments: https://youtu.be/--jpFMhez9g.

^19^The controller can be tested at this repository: https://github.com/graiola/wbc-setup.

## Supplementary Material

Supplementary Video 1 Experiments.

## Appendix

### Relating Inverse Dynamics to Impedance Control

This appendix will show how it is possible to relate inverse dynamics to Cartesian impedance control by opportunely selecting tasks gains.

Let us first consider a *naive* example with a single postural task:

(A1) $$\begin{array}{l} {\ddot{q}}^{*}=\underset{\ddot{q}}{\mathrm{argmin}}\;∥\ddot{q}-{\ddot{q}}_{r}∥, \end{array}$$

with solution:

(A2) $$\begin{array}{l} {\ddot{q}}^{*}={\ddot{q}}_{r}=\text{K}\left({\text{q}}_{d}-\text{q}\right). \end{array}$$

To neglect the inertia matrix, we just need to choose a particular gain for the ${\ddot{q}}_{r}$:

(A3) $$\begin{array}{l} {\ddot{q}}_{r}={\text{M}}^{-1}{\text{K}}^{\prime }\left({\text{q}}_{d}-\text{q}\right)={\text{M}}^{-1}\tau_{r}. \end{array}$$

If we plug (A3) in (3), neglecting non-linear terms, we obtain:

(A4) $$\begin{array}{l} \tau =M{\ddot{q}}_{r}=\tau_{r}, \end{array}$$

obtaining the classic joint impedance control where the inertia matrix does not appear.

We now consider the optimization in (13) for the unconstrained case of a single, full rank, Cartesian task:

(A5) $$\begin{array}{l} {\ddot{q}}^{*}=\underset{\ddot{q}}{\mathrm{argmin}}\;∥\text{J}\ddot{q}-{\ddot{x}}_{r}∥, \end{array}$$

with **J** ∈ ℝ^6×6^. If we compute the Lagrangian from (5) we obtain:

(A6) $$\begin{array}{l} L=\frac{1}{2}{\ddot{q}}^{T}{\text{J}}^{T}\text{J}\ddot{q}-{\left(\text{J}\ddot{q}\right)}^{T}{\ddot{x}}_{r}+{\ddot{x}}_{r}^{T}{\ddot{x}}_{r}. \end{array}$$

To solve (A5) we derive the Lagrangian:

(A7) $$\begin{array}{l} \frac{\partial L}{\partial \ddot{q}}={\text{J}}^{T}\text{J}\ddot{q}-{\text{J}}^{T}{\ddot{x}}_{r}=0. \end{array}$$

With the hypothesis of **J** full rank, the matrix **J**^*T*^**J**, is invertible and the solution of (A5) is given by:

(A8) $$\begin{array}{l} \ddot{q}={\left({\text{J}}^{T}\text{J}\right)}^{-1}{\text{J}}^{T}{\ddot{x}}_{r}={\text{J}}^{-1}{\ddot{x}}_{r}={\text{J}}^{-1}\left(\text{K}\left({\text{x}}_{d}-\text{x}\right)-\overset{\cdot }{J}\overset{\cdot }{q}\right). \end{array}$$

In this case, we can neglect the inertia imposing:

(A9) $$\begin{array}{c} {\ddot{x}}_{r}=\text{K}\left({\text{x}}_{d}-\text{x}\right)-\overset{\cdot }{J}\overset{\cdot }{q}=\text{J}{\text{M}}^{-1}{\text{J}}^{T}{\text{K}}^{\prime }\left({\text{x}}_{d}-\text{x}\right)-\overset{\cdot }{J}\overset{\cdot }{q}\\ =\Lambda^{-1}{\text{F}}_{r}-\overset{\cdot }{J}\overset{\cdot }{q}, \end{array}$$

where **Λ** = (**J****M**^−1^**J**^*T*^)^−1^ is the Cartesian inertia matrix of the task. (A9) plugged in (3) returns:

(A10) $$\begin{array}{l} \tau ={\text{J}}^{T}{\text{F}}_{r}-\text{M}{\text{J}}^{-1}\overset{\cdot }{J}\overset{\cdot }{q}. \end{array}$$

Notice that (A10) is equivalent to a Cartesian impedance controller (Ott, 2008).

Finally, let us consider the final level of a hierarchical controller. Again we consider the optimization in (13):

(A11) $$\begin{array}{l} \begin{array}{c} {\ddot{q}}^{*}=\underset{\ddot{\text{q}}}{\mathrm{argmin}}\;∥\ddot{q}-{\ddot{q}}_{r}{∥}_{\text{W}}\\ \text{subject to}\\ \;\text{J}\ddot{q}={\ddot{x}}^{*}, \end{array} \end{array}$$

with **J** ∈ ℝ^*m* × *n*^ containing all the Jacobians from the previous levels and ***ẍ***^*^ ∈ ℝ^*m*^ all the optimal accelerations obtained at the previous levels. The Lagrangian is given by:

(A12) $$\begin{array}{l} L=\frac{1}{2}{\ddot{q}}^{T}\text{W}\ddot{q}-{\ddot{q}}^{T}\text{W}{\ddot{q}}_{r}+{\ddot{q}}_{r}^{T}\text{W}{\ddot{q}}_{r}+\lambda^{T}\left(\text{J}\ddot{q}-{\ddot{x}}^{*}\right), \end{array}$$

which leads to the following optimal conditions:

(A13) $$\begin{array}{l} \begin{array}{c} \frac{\partial L}{\partial \ddot{q}}=\text{W}\ddot{q}-\text{W}{\ddot{q}}_{r}+{\text{J}}^{T}\lambda =0,\\ \frac{\partial L}{\partial \lambda }=\text{J}\ddot{q}-{\ddot{x}}^{*}=0. \end{array} \end{array}$$

The final optimal accelerations are given by:

(A14) $$\begin{array}{l} \ddot{q}*={\text{W}}^{-1}{\text{J}}^{T}{\left(\text{J}{\text{W}}^{-1}{\text{J}}^{T}\right)}^{-1}{\ddot{x}}_{r}^{*}\\ \;+{\left(\text{I}-{\text{W}}^{-1}{\text{J}}^{T}\left(\text{J}{\text{W}}^{-1}{\text{J}}^{T}\right)\text{J}\right)}^{-1}{\ddot{q}}_{r}, \end{array}$$

it is well known that is possible to achieve the dynamically consistent inverted Jacobian (Khatib, 1987) posing **W** = **M**:

(A15) $$\begin{array}{l} \begin{array}{c} \ddot{q}*={\bar{J}}^{†}{\ddot{x}}_{r}^{*}+\left(\text{I}-{\bar{J}}^{†}\text{J}\right){\ddot{q}}_{r}\\ {\bar{J}}^{†}={\text{M}}^{-1}{\text{J}}^{T}{\left(\text{J}{\text{M}}^{-1}{\text{J}}^{T}\right)}^{-1}. \end{array} \end{array}$$

We now plug (A3) and (A9) into (A15):

(A16) $$\begin{array}{l} \ddot{q}*={\text{M}}^{-1}{\text{J}}^{T}{\text{F}}_{r}-{\text{M}}^{-1}{\text{J}}^{T}\Lambda \overset{\cdot }{J}\overset{\cdot }{q}+\left(\text{I}-{\bar{J}}^{†}\text{J}\right){\text{M}}^{-1}\tau_{r} \end{array}$$

which plugged into (3) returns:

(A17) $$\begin{array}{l} \tau ={\text{J}}^{T}{\text{F}}_{r}-{\text{J}}^{T}\Lambda \overset{\cdot }{J}\overset{\cdot }{q}+\left(\text{I}-{\text{J}}^{T}{\left(\text{J}{\text{M}}^{-1}{\text{J}}^{T}\right)}^{-1}\text{J}{\text{M}}^{-1}\right)\tau_{r} \end{array}$$

which again corresponds to a Cartesian impedance controller with dynamically consistent null-space projection (Ott, 2008).
